# Local impedance drop–guided versus lesion size index–guided pulmonary vein isolation

**DOI:** 10.1007/s10840-024-01870-3

**Published:** 2024-07-12

**Authors:** Evgeny Lian, Robert Pantlik, Vera Maslova, Sven Willert, Fabian Moser, Andrew Remppis, Derk Frank, Thomas Demming

**Affiliations:** 1 Department of Internal Medicine III (Cardiology and Intensive Care Medicine), University Hospital Schleswig-Holstein (UKSH), Kiel, Germany; 2 Department of Cardiology, Heart and Vascular Center, Bad Bevensen, Germany

**Keywords:** Atrial fibrillation, Local impedance, Lesion size, Pulmonary vein isolation, Radiofrequency ablation

## Abstract

**Background:**

Local tissue impedance drop (LID) and lesion size index (LSI) technologies are valuable for predicting effective lesion formation. This study compares the acute and long-term efficacy of LID-guided versus LSI-guided pulmonary vein isolation (PVI) for atrial fibrillation treatment.

**Methods:**

We retrospectively analyzed two patient groups undergoing radiofrequency PVI. In the LID-guided group (*n* = 35), ablation was performed without contact force monitoring, stopping at the LID plateau (target LID 12 Ohm posterior, 16 Ohm anterior). In the LSI-guided group (*n* = 31), ablation used contact force information with target LSI (5 anterior, 4 posterior). Both groups utilized a power of 40 W anterior and 30 W posterior, with < 6 mm inter-lesion distance. Gap mapping and touch-up ablation were done if necessary.

**Results:**

PVI was achieved with a significantly shorter ablation time in the LSI-guided group (25 min [21;31] vs 30 [27;35], *p* = 0.035). PV gaps were more frequent in the LID-guided group (74% vs 42%, *p* = 0.016). Over 11.5 ± 2.9 months follow-up, arrhythmia recurrence was higher in the LID-guided group (34.3% vs 16.1%, *p* = 0.037). A redo procedure performed in 10 (28.6%) patients in the LID-guided group and 3 (9.7%) in the LSI-guided group showed chronic PV reconnections in 7 out of 10 (70%) and 2 out of 3 (67%) patients, respectively.

**Conclusions:**

LSI-guided ablation results in shorter ablation time and fewer PV gaps compared to LID-guided ablation. Despite initial success, LID-guided ablation had higher arrhythmia recurrence and PV reconnections during long-term follow-up compared to LSI-guided ablation.

**Graphical abstract:**

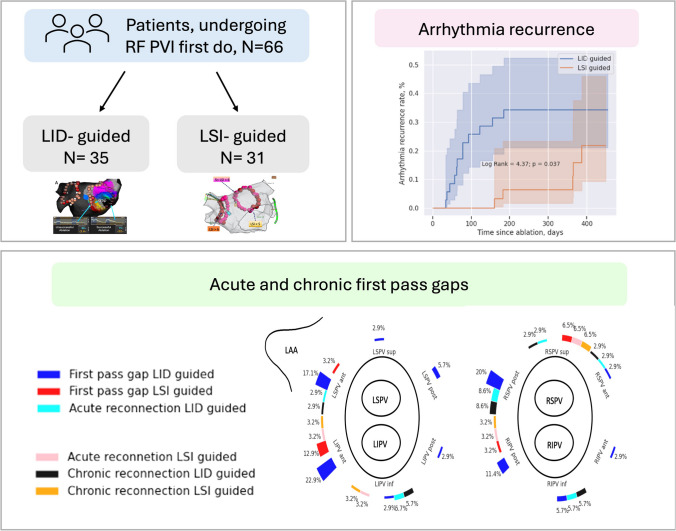

**Supplementary Information:**

The online version contains supplementary material available at 10.1007/s10840-024-01870-3.

## Introduction

Pulmonary vein isolation (PVI), as a well-established cornerstone of catheter ablation of atrial fibrillation (AF), depends on continuous, durable circular lesions around the pulmonary vein (PV) antra [1]. Electrical reconduction between the PV and the atrial myocardium is the primary cause of AF recurrence in patients after catheter ablation [2].

Therefore, predicting lesion size is critical for a successful and safe procedure.

Lesion Size Index (LSI, EnSite™, Abbott) using catheter contact force (CF) monitoring and local impedance monitoring (LI, DirectSence™ Technology, Boston Scientific) dynamic are methods to control the quality of the catheter-tissue interface and the efficacy of radiofrequency (RF) ablation [3, 4]. Both methods have shown their feasibility when used for radiofrequency PVI. The LSI algorithm uses CF, duration of RF energy delivery, and electrical current to calculate the lesion volume. Targeting LSI ≥ 5 results in shorter procedure, RF, fluoroscopy times, and fewer touch-up ablations [5].

DirectSense™ technology is based on monitoring the LI dynamics to assess the quality of the catheter contact with the tissue and estimate the RF ablation duration [4, 6–8]. LI increases and reaches its plateau as the contact pressure of the catheter to the tissue increases [4]. LI decreases immediately after the start of the RF delivery and reaches its plateau at the end of the resistive heating phase. By stopping RF delivery at the LI drop plateau, the conductive heating phase of lesion formation can be avoided. The localized trial demonstrated that LID-guided PVI can be feasible and effective even without contact force measurement [9]. The investigators defined LI drop values of 16.1 ohms for anterior segments and 12.3 ohms for posterior segments as predictive of durable lesions.

It is unclear whether the stand-alone non-CF-based LI-guided ablation is as effective and safe as the CF-based technology for durable PVI, as neither technology has been directly compared.

This study compares the acute and chronic efficacy of non-CF-based LID-guided versus CF-based LSI-guided PVI.

## Methods

### Patient population

In this retrospective, non-randomized, double-arm comparative study, we enrolled consecutive patients with symptomatic drug-refractory AF who underwent a de novo radiofrequency PVI procedure using either a local impedance drop (LID-guided group) or a lesion size index (LSI-guided group) to predict lesion formation. The study was conducted using the tenets of the Declaration of Helsinki. The local ethics committee approved the study protocol, and all patients gave written informed consent.

### Pulmonary vein isolation procedure

All procedures were carried out under conscious sedation. A diagnostic catheter was placed in the coronary sinus as an electrical reference. A heparin bolus was administered before the double transseptal puncture to maintain an activated clotting time greater than 300 s. Selective PV angiography was performed to mitigate the difference in the anatomical representation of PV anatomy between the mapping systems, followed by the tagging of PV ostia. We performed high-density mapping of the left and PVs in SR or during ongoing AF to define the anatomy and bipolar voltage characteristics of the chambers, using a 64-pole basket diagnostic catheter (Orion™) with Rhythmia 3D navigation system (Boston Scientific) for LID-guided group and a 16-pole grid catheter (HD Grid™) with Ensite Precision 3D-navigation system (Abbott laboratories) for the LSI-guided group. Each PV antrum was divided into six anatomical segments (12 per patient) for further assignment of ablation point locations and gap localization.

**Fig. 1:**
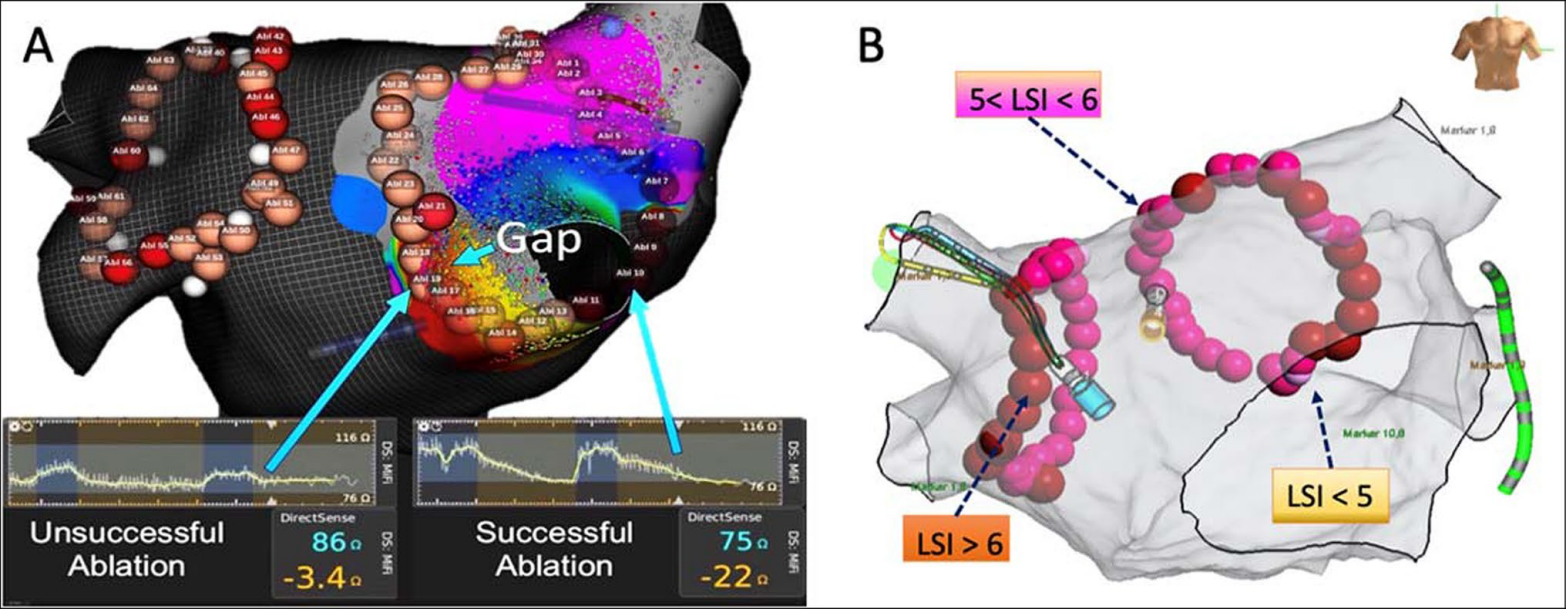
Representative examples of 3D maps of the left atrium with antral circumferential PVI. **A** Local impedance traces during LID-guided ablation. **B** LSI-guided ablation. LID, local tissue impedance drop; LSI, local size index

First-pass antral encircling of the ipsilateral PVs with roughly at least 1 cm distance from the PV ostia was performed using point-by-point ablation. In the LID-guided group (*n* = 35), energy was delivered using an IntellaNav MiFi™ (Boston Scientific, MA, USA) ablation catheter without CF monitoring. The quality of the contact was assessed solely based on the LI dynamics. LID target was 16 Ohm at the posterior and 12 Ohm at the anterior segments. Ablation was terminated when LID reached a plateau (Fig. 1A). In the LSI-guided group (*n* = 31), lesions were created using Tacticath™ (Abbott, MA, USA) ablation catheter with continuous CF monitoring until the target LSI was reached at each point (LSI = 5 for anterior and LSI = 4 for posterior segments respectively, Fig. 1B). An inter-lesion distance of < 6 mm and a power of 40 W for anterior and 30 W for posterior segments was used in both groups.

Assessment and mapping of residual conduction gaps followed by touch-up ablation were performed along the index ablation line if first-pass encircling PVI was not achieved or in the case of an acute reconnection after a 20-min waiting period. AF, persisting after PVI, was terminated with electrical cardioversion. Pulmonary vein isolation was confirmed by entrance block in all cases. No additional ablations at the carina regions or in the LA were performed. All antiarrhythmic drugs, except beta-blockers, were discontinued after ablation if patients received them before the procedure.

#### Follow-up and redo procedure

Long-term follow-up included a Holter ECG at 3, 6, and 12 months after the initial ablation and symptom-driven visits with ECG registration. In case of symptomatic arrhythmia recurrence, a second procedure was performed, including validation of PVI and mapping of the reconnection site followed by RePVI, if necessary.

### Statistical analysis

**Fig. 2:**
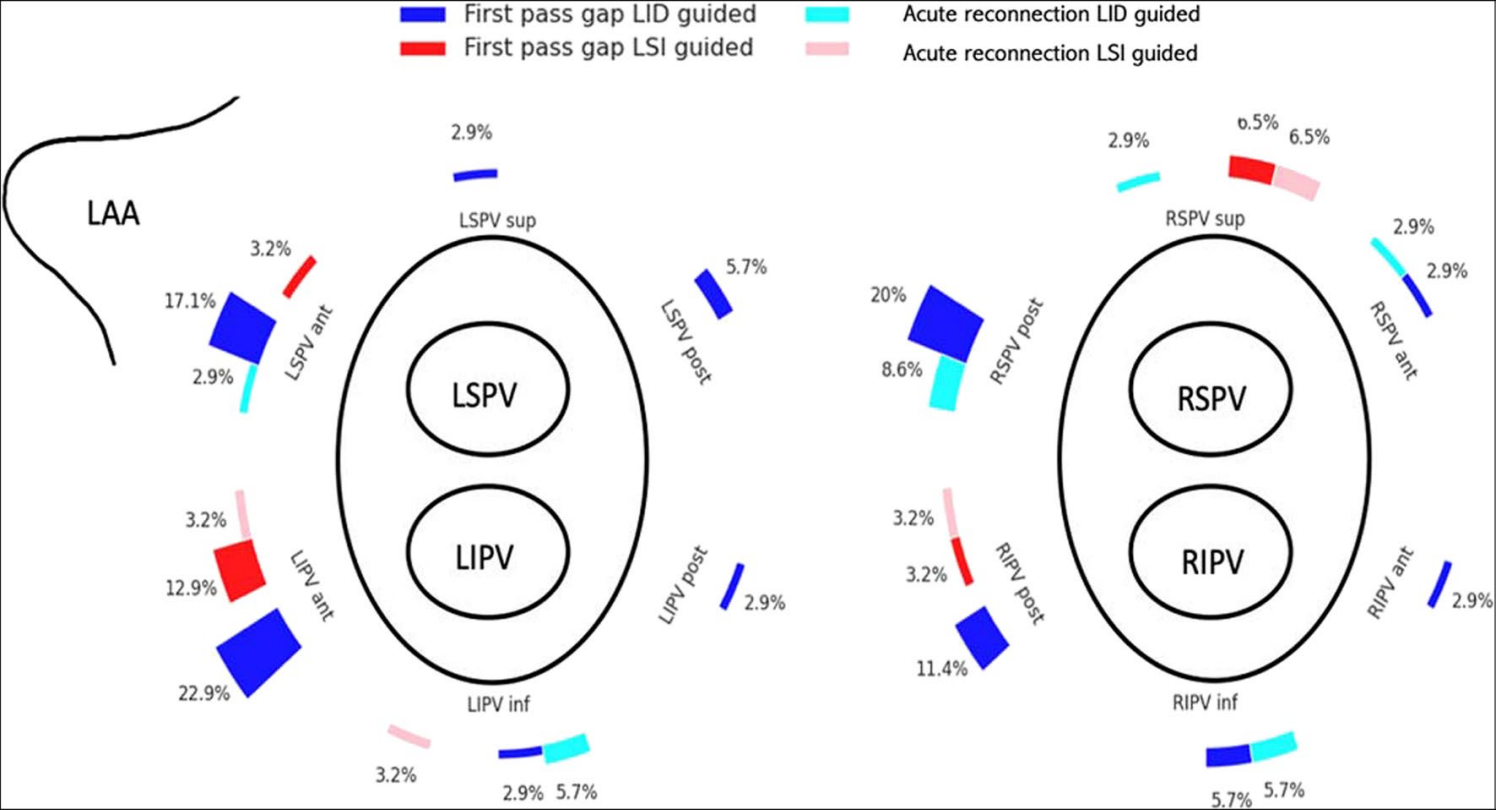
Distribution of acute PV gaps in different segments of PV antra. LAA, left atrial appendage; LIPV, left inferior pulmonary vein; LSPV, left superior pulmonary vein; RIPV, right inferior pulmonary vein; RSPS, right superior pulmonary vein; PV, pulmonary vein

Radiofrequency (RF) tags 3-dimensional locations with filtered local impedance, system impedance, CF, signal amplitude, LSI, ablation duration, and power were processed offline using Python 3 programming language (SciPy and Sklearn packages). Initial LI and system impedance (SI) and LI drop and SI drop were calculated from the filtered data for each ablation lesion. Ablation points were assigned to the corresponding segments of the PV antrum (Fig. 2). The median LID, minimum LID, median system impedance drop, CF, and LSI values were calculated for each segment. The maximum inter-lesion distance was calculated for each segment to characterize the largest distance between RF applications. Continuous data are presented as mean ± standard deviation; skewed continuous parameters were expressed as median (interquartile range defined as Q1–Q3). Categorical data were summarized as frequencies and percentages. Comparisons between baseline characteristics were performed using Student’s *t*-test, Mann–Whitney rank-sum, *χ*^2^, or Fisher’s exact tests, as appropriate. We used ROC analysis to define the predictive values of different thresholds of intraprocedural parameters for the detection of gap formation. A two-tailed *p* < 0.05 was considered statistically significant. Kaplan–Meier survival analysis was used to estimate and compare the time to arrhythmia recurrence between the LID-guided and LSI-guided groups. The difference in survival probabilities was tested for statistical significance using the log-rank test.

## Results

Sixty-six patients with documented AF were included, who underwent either LID-guided (35 patients) or LSI-guided PVI (31 patients). The clinical characteristics of the patients are shown in Table 1. There was no statistically significant difference in baseline characteristics between the two groups, including the number of patients with persistent AF (*n* = 24; 68.6% in the LID-guided group and *n* = 23; 74.2%, *p* = 0.82).

**Table 1:** Patient’s baseline characteristics

	LID-guided PVI (*n* = 35)	LSI-guided PVI (*n* = 31)	p*
Age (years)	67 (63–70)	62(58–70)	0.29
Male, *n* (%)	20 (57.1)	21 (67.7)	0.53
BMI	28 (27–32)	29 (26–31)	0.85
LA diameter (mm)	42 (38–46)	42 (38–44)	
Ejection fraction, %	55 (53–60)	55 (50–59)	0.38
Persistent AF, *n* (%)	24 (68.6)	23 (74.2)	0.82
Concomitant atrial flutter, *n* (%)	6 (17.1)	7 (22.6)	0.81
Hypertension, *n* (%)	32 (91.4)	27 (87.1)	0.87
Ischemic heart disease, *n* (%)	8 (22.9)	6 (19.4)	0.96
Heart failure, *n* (%)	7 (20)	11 (35.5)	0.26
NYHA Class I, *n* (%)	1 (2.9)	1 (3.2)	0.34
NYHA Class II, *n* (%)	5 (14.3)	6 (19.4)	
NYHA Class III, *n* (%)	1 (2.9)	4 (12.9)	
Diabetes Mellitus, *n* (%)	5 (14.3)	2 (6.5)	0.53
CHA_2_DS_2_-VASc score	3 (2–3)	2 (2–3)	0.35
Class I AAD, *n* (%)	5 (14.3)	1 (3.2)	0.24
Class III AAD, *n* (%)	7 (20)	5 (16.1)	
beta-Blockers, *n* (%)	23 (65.7)	25 (80.6)	
DOAC, *n* (%)	31 (88.6)	29 (93.5)	0.78
VKA, *n* (%)	4 (11.4)	2 (6.5)	

*LID*, local impedance drop; *LSI*, lesion size index; *PVI*, pulmonary vein isolation; *BMI*, body mass index; *LA*, left atrial; *AF*, atrial fibrillation; *NYHA*, New York Heart Association; *AAD*, antiarrhythmic drugs; *DOAC*, direct oral anticoagulant; *VKA*, vitamin K antagonist

### Procedural data

All PVs (264 out of 264) were successfully isolated in each patient by encircling the ipsilateral veins. Median ablation time was significantly longer in the LID-guided group (30 min; IQR 27–35 min) than in the LSI-guided group (25 min; IQR 21–31 min; *p* = 0.035). There was no statistically significant difference in total procedure time and fluoroscopy time between groups (Table 2). No major complications occurred during the procedure or at follow-up.

**Table 2:** Procedural characteristics, incidence of pulmonary vein gaps, and follow-up data

	LID-guided	LSI-guided	*p*-value
Number of patients, *n*	35	31	
Number of circles around PV antra, *n*	70	62	
Procedure time, min	150 (124–240)	156 (137–182)	0.31
Fluoroscopy time, min	12 (7.7–16)	11 (6.2–15)	0.25
RF ablation time, min	30 (27–35)	25 (21–31)	0.035
First-pass gaps			
Number of first-pass gaps, *n* (per patient)	34 (1.0 ± 1.1)	7 (0.2 ± 0.4)	< 0.001
PV antra with a first-pass gap, *n* (%)	27 (39)	8 (13)	0.002
Patients with a first-pass gap, *n* (%)	20 (57)	7 (23)	0.009
Acute reconnections			
Number of acute reconnections, *n* (per patient)	10 (0.3 ± 0.6)	7 (0.1 ± 0.3)	0.19
PV antra with acute PV reconnections, *n* (%)	9 (13)	5 (8)	0.54
Patients with acute PV reconnections, *n* (%)	8 (23)	4 (13)	0.46
Overall gaps during the index procedure			
Total PV antra with gaps, *n* (%)	37 (53)	12 (19)	< 0.001
Total patients with PV gaps, *n* (%)	26 (74)	13 (42)	0.016
Arrhythmia recurrence during follow-up			
Patients with arrhythmia recurrence, *n* (%)	12 (34.3)	5 (16.1)	0.09
AF recurrence, *n* (%)	9 (25.7)	4 (12.9)	0.55
AT/AFL recurrence, *n* (%)	3 (8.6)	2 (6.4)	0.55
ReDo procedure			
ReDo ablations performed	10 (28.6)	3 (9.7)	0.11
Patients with chronic PV reconnections, *n* (%)	7/10 (70)	2/3 (67)	1.0

*LID*, local impedance drop; *LSI*, lesion size index; *PV*, pulmonary veins; *RF*, radiofrequency; *AF*, atrial fibrillation; *AT*, atrial tachycardia; *AFL*, atrial flutter

### Characterization of local impedance (LI) in the LID-guided group.

The mean baseline LI, mean LID, baseline SI, mean SI drop, and gap rate for each of the 12 anatomical segments measured in the LID-guided group are shown in Supplementary Table 1. A significantly higher mean baseline LI (105.4 ± 13.0 vs 100.7 ± 14.0 ohms, *p* < 0.0001), as well as deeper LID (13.9 ± 9.6 vs 12.6 ± 8.5 ohms, *p* = 0.002), was found in the right PVs, as compared to the left PVs.

Despite the higher mean baseline SI in the right PVs segments, as compared to the left PVs segments (108.3 ± 16.0 vs. 104.1 ± 15.4 ohms, *p* < 0.0001), the mean SI drop (6.6 ± 6.4 vs 6.2 ± 6.4 ohms, *p* = 0.271) was not statistically different between the right and left PVs.

The mean baseline LI (103.5 ± 13.8 vs 96.9 ± 10.7 ohms, *p* < 0.0001) and LI drop (13.8 ± 9.1 vs 5.3 ± 3.1 ohms, *p* < 0.0001) were significantly higher in successful sites as compared to first-pass gap or acute reconnection sites. The correlation between LI and SI drop (*r* = 0.45, *p* < 0.0001) was modest (Fig. 4D in the Supplement). The predefined LID target of 16 Ohm for anterior and 12 Ohm for posterior segments was met only in 22.4% and 55.1%, respectively.

### Lesion size index and system impedance in the LSI-guided group

In the LSI-guided group, the mean target LSI of 5 for the anterior segments and 4 for the posterior segments was achieved in all antral segments except the anterior part of the left PVs (Supplementary Table 2). Most of the gaps were observed in the inferior-anterior segment of the left PVs (33.3% of all gaps in the LSI-guided group). The LSI was significantly lower in the points with the gaps than those without (3.8 ± 1.0 vs 4.9 ± 1.1, *p* < 0.0001). The mean baseline SI (115.7 ± 12.7 vs 116.4 ± 8.1 ohms, *p* < 0.7311) was not statistically different between successful points and points with gaps. On the other hand, SI drop magnitude was significantly higher in the successful points compared to the points with the first-pass gap or acute reconnection (14.3 ± 6.4 vs 12.0 ± 4.4 ohms, *p* < 0.029). The correlation between LSI and SI drop (*r* = 0.08, *p* < 0.0001) was weak (Fig. 4(C) in the Supplement).

### Group comparison

#### First-pass gaps

PVI by first-pass encirclement was achieved in a much smaller proportion of PV circles in the LID-guided group (43 out of 70 [61%] circles) as compared to the LSI-guided group (54 out of 62 [87%] circles, *p* = 0.002). After the first pass encircling, a significantly higher number of gaps were observed in the LID group, which were identified and closed by touch-up ablation (34 gaps in 20 out of 35[57%] patients in the LID-guided group vs 7 gaps in 7 out of 31 [23%] patients in LSI-guided group, *p* = 0.009).

#### Acute reconnections after 20 min of waiting time

The difference in the rate of acute reconnections was not statistically significant (LID-guided 9 out of 70 [13%] circles vs. LSI-guided 5 out of 62 [8%] circles, *p* = 0.54). Overall, significantly more patients had PV gaps (first-pass gaps and acute reconnections) when LID-guided ablation was performed as compared to LSI-guided ablation (26 out of 35[74%] vs 13 out of 31 [42%] respectively, *p* = 0.016).In the post hoc analysis, a LID < 9.7 ohms was the only predictive parameter for the PV gap (sensitivity = 96.4%, specificity = 67%, AUC = 0.85) in the LID-guided group (Fig. 5 in Supplement).

For the paroxysmal AF subgroups, a statistically significant difference was observed in the proportion of the patients with PV-gaps (9 out of 11 [81.8%] paroxysmal AF patients in the LID-guided group and 2 out of 8 [25%] in the LSI-guided group, *p* = 0.045).

#### Arrhythmia recurrence

All patients remained off antiarrhythmic therapy except the beta-blockers during the 11.5 ± 2.9 months follow-up. Twelve out of 35 (34.3%) patients from the LID-guided group and 5 out of 31 (16.1%) patients from the LSI-guided group experienced arrhythmia recurrence (Table 2). In the LID-guided group, 9 (25.7%) patients had AF, 2 (5.7%) left atrial reentry tachycardia, and 1 (2.9%) typical atrial flutter. In the LSI-guided group, 4 (12.9%) patients had AF, 1 (3.2%) left atrial reentry tachycardia, and 1 (3.2%) typical atrial flutter. There was no statistically significant difference between the study groups regarding arrhythmia recurrence type. In the LDI-guided group, 3 out of 11(27.3%) patients with PAF had AF recurrence, whereas in the LSI-guided group, 1 out of 8 (12.5%) PAF patients experienced AF recurrence (*p* = 0.83).

**Fig. 3:**
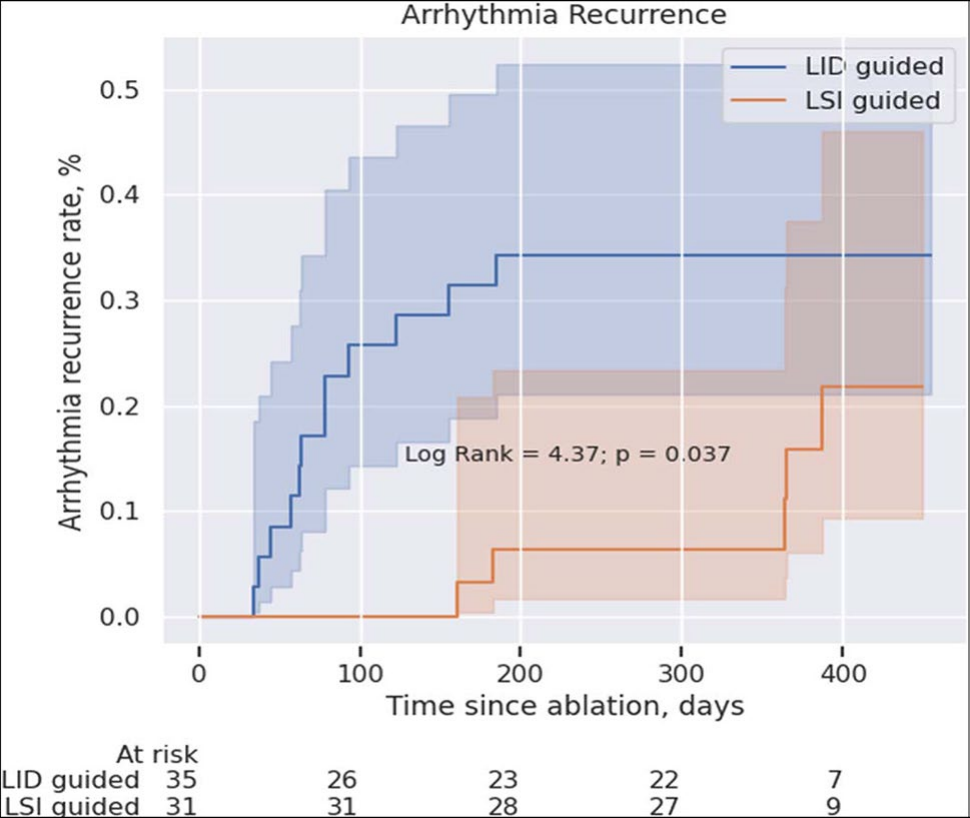
Kaplan–Meier curves comparing arrhythmia recurrence in the two trial groups. LID, local tissue impedance drop; LSI, local size index

Kaplan–Meier survival analysis showed a statistically significant higher probability of arrhythmia recurrence in patients treated with the LID-guided approach (log-rank = 4.37, *p* = 0.037, Fig. 3). Redo procedures were performed in 10 (28.6%) patients in the LID-guided group and 3 (9.7%) patients in the LSI-guided group, and chronic pulmonary vein reconnections were detected in 7 out of 10 (70%) and 2 out of 3 (67%) patients, respectively (Fig. 2).

## Discussion

Our study provides the first direct comparison of non-CF-based LID-guided ablation technology with CF-based LSI-guided. We report that LID-guided ablation has a higher incidence of first-pass gaps and acute reconnections, requiring longer energy delivery and additional ablations.

The optimal timing of initiation and termination of RF energy delivery is critical in cardiac ablation to ensure lesion efficacy and minimize reversible damage [10] The catheter-tissue interface must be thorough at the start of energy delivery to avoid reversible lesion formation. The energy delivery should be long enough to achieve a permanent transmural lesion without compromising the ablation safety. In the past, the decision to start and stop energy delivery relied on the interventionalist’s judgement or an arbitrary ablation time. However, with LSI and DirectSense™ technologies, we now have more refined methods to assess the catheter-tissue interface quality and the optimal energy delivery duration for each ablation site [9, 11].

Even though the LI-guided non-CF-based ablation has been shown to be highly effective [9], compared to the CF-based ablation, it showed lower reliability in estimating the catheter-tissue interface prior to each RF application compared to the stand-alone LI monitoring. For instance, edema from previously delivered nearby lesions may affect the accuracy of LI as a surrogate for contact because the impedance of unabated tissue will be lowered just by the presence of edema. Many factors can influence the LI dynamic: contact, edema after adjacent RF applications, and fibrotic or fat tissue. While LI monitoring provides valuable insight into the dynamics of lesion formation, it cannot prevent the development of edema due to insufficient ablation attempts when initial contact with the tissue is inadequate. The estimation of the contact by the dynamic of initial LI, reported by Das et al. and adopted in our study, probably was not so precise enough in our patient population to reproduce the values of the LOCALIZE trial. In our study, we could not reach the targeted values of LID in most of the RF applications. Despite acute PVI in all patients, edema formation led to a higher rate of chronic PV reconnections and subsequent AF recurrence observed in the LID-guided ablation group.

Moreover, the presence of fibrosis in our mostly persistent AF patient population may result in lower LID as compared to the LOCALIZE trial results with the paroxysmal AF population. Still, it does not explain the superiority of the LSI-guided ablation, as the LID-guided and LSI-guided groups were comparable regarding the AF type. Therefore, the clinical significance of our findings is twofold: It highlights the potential for improved patient outcomes through the integration of CF monitoring, and it calls for a re-evaluation of the role of stand-alone LI-guided ablation.

Recently, CF technology has been combined with LI monitoring in the new ablation catheter (StablePoint™, Boston Scientific), but no lesion size index has been developed [12]. A recent ex vivo study showed that lesions guided by LI were larger with the CF-sensing StablePoint catheter relative to the MiFi-OI ablation catheter [13]. While this new platform may improve the efficacy and safety of ablation, the studies comparing CF-based LSI-guided ablation and LID-guided ablation combined with CF monitoring are limited. Moreover, recently introduced new ablation catheter platforms with optimized tip irrigation allow “high power short duration” RF application, which may increase the effectiveness and shorten the procedure time [14, 15].

## Study limitations

This study has several limitations that should be considered when applying the results to clinical practice. First, this is a retrospective, non-randomized analysis with a low number of patients. Second, only patients with symptomatic AF recurrence underwent redo ablation. Therefore, the actual rate of chronic PV reconnection cannot be determined. As we used ablation catheters without incorporating CF technology for LID-guided ablation, we cannot conclude whether the higher rate of PV gaps is influenced by poor contact or whether the resistive heating phase is insufficient for transmural lesion formation. CF-sensing catheters measuring LI have become available, and future studies are required to elucidate whether the comparative benefit was driven by LSI as a lesion quality marker versus utilizing contact force.

## Conclusion

LSI-guided CF-based ablation resulted in successful PVI with significantly shorter ablation times and fewer PV gaps than LID-guided non-CF-based ablation. Despite initial successful isolation of all PVs, LID-guided ablation resulted in a considerably higher probability of arrhythmia recurrence during long-term follow-up and a higher rate of chronic PV reconnections than LSI-guided ablation. This reflects the superior nature of direct CF monitoring over the local impedance information for anticipating the quality of the catheter-tissue interface before each application.

## Supplementary Information

Below is the link to the electronic supplementary material.

Supplementary file1 (DOCX 1693 KB)
