# Supplementary material for: Local impedance drop–guided versus lesion size index–guided pulmonary vein isolation
Source: J Interv Card Electrophysiol. 2024 Jul 12;67(9):2051–8. doi: 10.1007/s10840-024-01870-3 (PMC11711155; doi:10.1007/s10840-024-01870-3)
Supplement: Supplementary file 1 — Supplementary file1 (DOCX 1693 KB) [file 10840_2024_1870_MOESM1_ESM.docx]

**Supplementary table 1.** Mean local impedance, system impedance and acute reconnection rates in different segments of pulmonary veins in LID-guided ablation group.

|  | Segment | Baseline LI(Ohm) | LI Drop (Ohm) | Baseline SI(Ohm) | SI Drop (Ohm) | RF duration (s) | Gap distibution(%) |
| --- | --- | --- | --- | --- | --- | --- | --- |
| Right |  | 105.4±13.0 | 13.9±9.6 | 108.3± 16.0 | 6.6±6.4 | 31.7±9.3 | 8.3 |
| 1 | Anterior superior | 108.8±14.9 | 19.4±10.6 | 109.2±17.1 | 8.2±6.2 | 32.5±10.1 | 5.7 |
| 2 | Anterior inferior | 106±12.2 | 19.1±9.9 | 109.8±16.7 | 8.3±6.5 | 31.8±8.8 | 2.9 |
| 3 | inferior | 104.5±11.9 | 14.9±8.9 | 108.9±16.8 | 6.6±7 | 31.6±9.9 | 11.4 |
| 4 | Posterior inferior | 104.4±11.9 | 11.1±7.7 | 107.9±15.2 | 5.8±6.5 | 32.2±9.7 | 11.4 |
| 5 | Posterior superior | 103.3±12.2 | 9.7± 6.7 | 107.9±15.1 | 5.4±5.2 | 31.8±8.8 | 28.6 |
| 6 | superior | 105.4±13.5 | 10.8±8.3 | 106.8±15.2 | 5.4±6.4 | 30.3±8.6 | 2.9 |
| Left |  | 100.7±14.0 | 12.6±8.5 | 104.1±15.4 | 6.2±6.4 | 31.3± 9.6 | 8.4 |
| 7 | Posterior superior | 104.3±16 | 11.7±7.1 | 107.9±15.3 | 6.4±6.3 | 31.7±7.1 | 5.7 |
| 8 | Posterior inferior | 99.1±9.6 | 10.2±6 | 104.1±15.5 | 6.2±6.2 | 31.1±9.1 | 2.9 |
| 9 | inferior | 97.3±13.4 | 12.1±10.7 | 102.3±15.4 | 5.6±6.5 | 32.4±10.3 | 8.6 |
| 10 | Anterior inferior | 98.9±15.2 | 13.3±10 | 101.8±15.7 | 6.0±7.3 | 32.5±11.0 | 22.9 |
| 11 | Anterior superior | 98±15.3 | 13.3±15.3 | 101.7±14.1 | 6.4±7.6 | 32.0±11.0 | 20.0 |
| 12 | superior | 104.8±15.9 | 14.5±8.8 | 106.2±15.3 | 6.6±5.4 | 28.8±8.4 | 2.9 |
|  | Overall | 103.1±13.7 | 13.3±9.1 | 106.3±15.8 | 6.4±6.4 | 31.5±9.5 | 8.3 |

LID: local impedance drop, LSI: lesion size index, RF, radiofrequency; SI, system impedance

**Supplementary table 2.** Mean LSI, system impedances and acute reconnection rates at different segments of pulmonary veins in the LSI-guided group.

|  | Segment | LSI | Mean CF | Baseline SI(Ohm) | SI Drop (Ohm) | RF duration (s) | Gap distribution (%) |
| --- | --- | --- | --- | --- | --- | --- | --- |
| Right |  |  |  |  |  |  |  |
| 1 | Anterior superior | 5.5±1.0 | 15.1±6.9 | 119.1±13.5 | 17.2±7.7 | 22.3±12.8 | 0 |
| 2 | Anterior inferior | 5.6±0.9 | 15.3±6.4 | 119.1±15.4 | 16.2±6.7 | 20.5±10.2 | 0 |
| 3 | inferior | 4.8±1.1 | 14.4±9.1 | 119.1±12.3 | 14.7±6.0 | 18.1±9.6 | 10.3 |
| 4 | Posterior inferior | 4.7±0.9 | 13.7±7.7 | 116.8±11.6 | 13.1±4.7 | 17.1±8.9 | 5.1 |
| 5 | Posterior superior | 4.8±1.0 | 13.8±7.7 | 117.9±12.2 | 13.5±5.6 | 17.8±9.5 | 7.7 |
| 6 | superior | 4.8±1.1 | 12.5±8.7 | 117.6±11.1 | 14.4±6.8 | 19.5±11.4 | 20.5 |
| Left |  |  |  |  |  |  |  |
| 7 | Posterior superior | 4.9±0.9 | 16.2±8.5 | 119.7±14.8 | 15.1±7.4 | 17.0±8.3 | 0 |
| 8 | Posterior inferior | 4.9±1.0 | 15.9±8.4 | 111.7±10.1 | 11.9±4.6 | 16.0±8.5 | 2.6 |
| 9 | inferior | 4.8±1.1 | 11.6±7.3 | 113.5±11.7 | 13.9±6.1 | 18.3±12.7 | 15.4 |
| 10 | Anterior inferior | 4.4±1.2 | 7.8±5.7 | 111.1±12.0 | 13.5±5.4 | 19.7±15.1 | 33.3 |
| 11 | Anterior superior | 4.6±1.2 | 9.6±7.3 | 113.3±11.5 | 14.1±6.6 | 20.2±15.4 | 2.6 |
| 12 | superior | 4.8±1.1 | 13.1±9.7 | 114.9±11.6 | 14.9±7.0 | 15.9±10.6 | 2.6 |

CF, contact force; LSI, lesion size index; RF, radiofrequency; SI, system impedance.

| *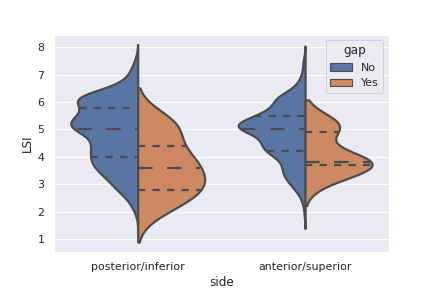*  A | *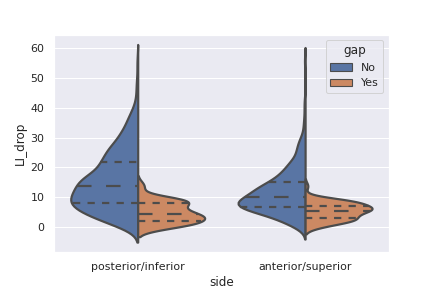*  B |
| --- | --- |
| 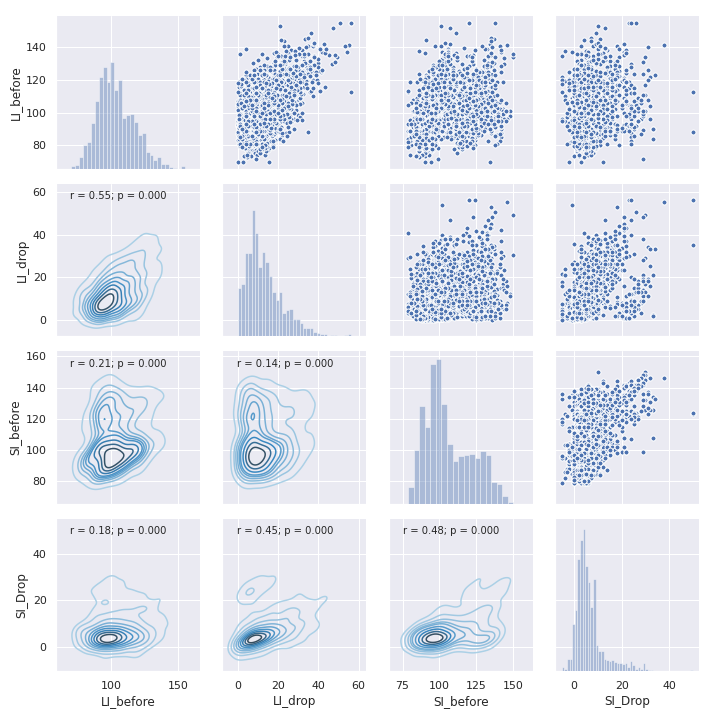  C | 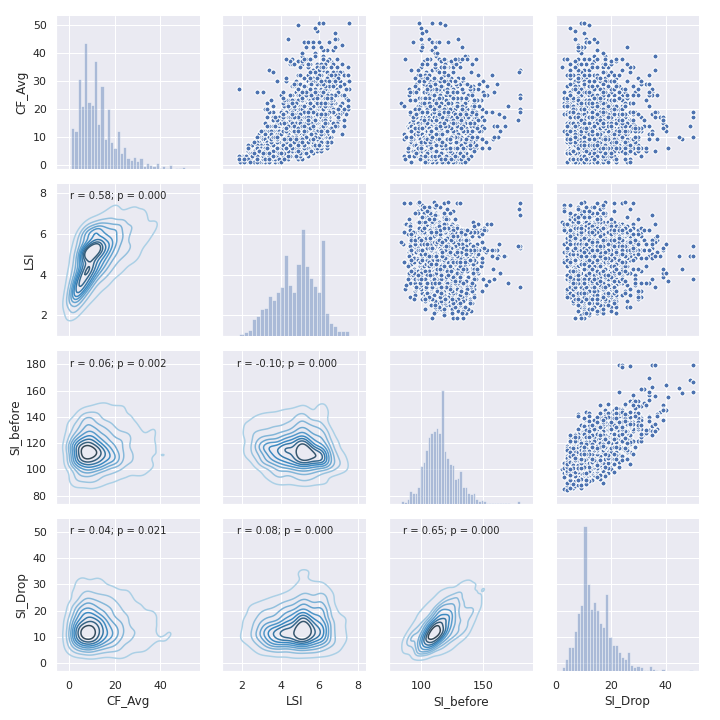  D |

**Supplementary Figure 1.** *(A)* Violine plot of comparing LSI value at the posterior and anterior segments of the antra in the successful ablation points and points with PV gaps. *(B)* Violine plot of comparing local impedance drop value at the posterior and anterior segments of the antra in the successful ablation points and points with PV gaps. *(C)* Scatter plots represent the correlation between the starting local and system impedance and local and system impedance drop observed in the local impedance drop-guided ablation group. *(D)* Scatter plots represent the correlation between the average CF, lesion size index, starting system impedance, and system impedance drop in the LSI-guided ablation group.

CF, contact force; LID, local tissue impedance drop; LSI, local size index


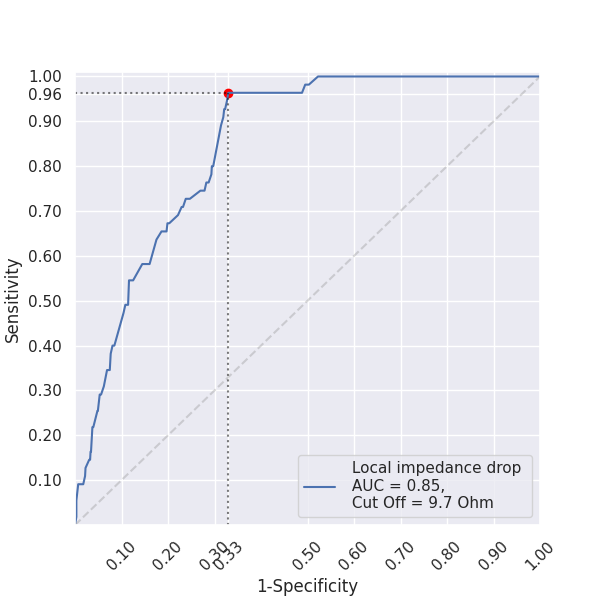


**Supplementary figure 2**. ROC-curve analysis of local impedance drop. The threshold value of optimal local impedance drop corresponds to the durable lesion.


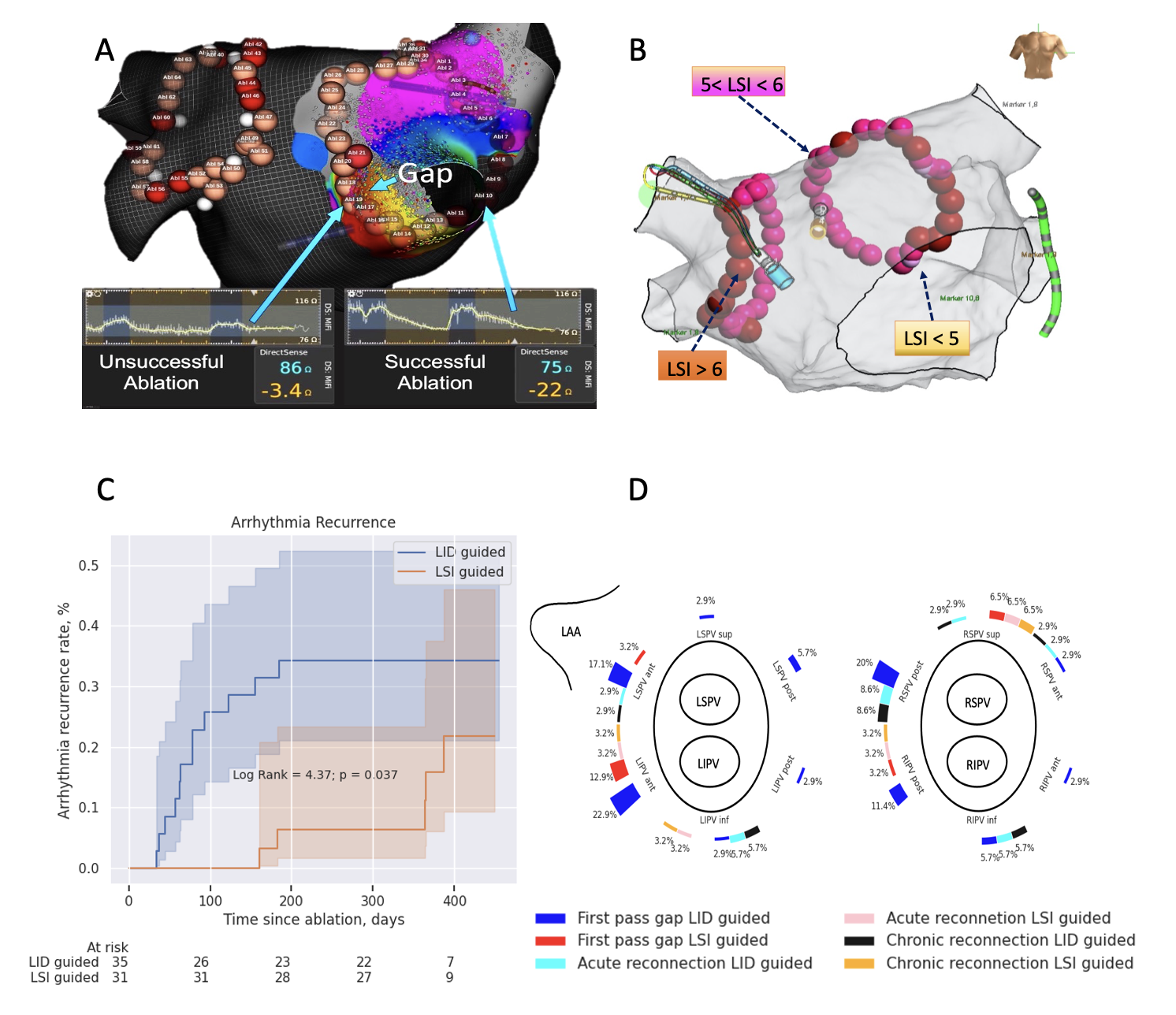


**Supplementary figure 3**. Distribution of acute and chronic PV gaps in different segments of PV antra.

LAA, left atrial appendage; LIPV, left inferior pulmonary vein; LSPV, left superior pulmonary vein; RIPV, right inferior pulmonary vein; RSPS, right superior pulmonary vein; PV, pulmonary vein
